# Silver Nanoparticles Proved to Be Efficient Antivirals In Vitro against Three Highly Pathogenic Fish Viruses

**DOI:** 10.3390/v15081689

**Published:** 2023-08-03

**Authors:** Andor Doszpoly, Mohamed Shaalan, Mansour El-Matbouli

**Affiliations:** 1 Veterinary Medical Research Institute, 21 Hungária krt., H-1143 Budapest, Hungary; 2 Department of Pathology, Faculty of Veterinary Medicine, Cairo University, Giza 12211, Egypt; 3 Division of Fish Health, Clinic for Avian and Fish Medicine, University of Veterinary Medicine, 1210 Vienna, Austria; mansour.el-matbouli@vetmeduni.ac.at

**Keywords:** silver nanoparticles, antiviral effect, in vitro assay, European catfish virus, spring viraemia of carp virus, Ictalurid herpesvirus 2

## Abstract

The efficacy of silver nanoparticles (AgNPs) was tested in vitro against three different fish viruses, causing significant economic damage in aquaculture. These viruses were the spring viraemia of carp virus (SVCV), European catfish virus (ECV), and Ictalurid herpesvirus 2 (IcHV-2). The safe concentration of AgNPs that did not cause cytotoxic effects in EPC cells proved to be 25 ng/mL. This dose of AgNPs decreased significantly (5–330×) the viral load of all three viruses in three different types of treatments (virus pre-treatment, cell pre-treatment, and cell post-treatment with the AgNPs). In a higher concentration, the AgNPs proved to be efficient against ECV and IcHV-2 even in a delayed post-cell-treatment experiment (AgNP treatment was applied 24 h after the virus inoculation). These first in vitro results against three devastating fish viruses are encouraging to continue the study of the applicability of AgNPs in aquaculture in the future.

## 1. Introduction

Nanoparticles are naturally occurring or engineered particles with a diameter ranging from 1 to 100 nm [1]. Extensive research on the applications of different nanoparticle types in the agricultural and veterinary sector has been conducted [2]. The applications vary from early diagnosis [3] and immunostimulant agents [4,5,6] to antimicrobial therapy [7,8,9].

Metallic nanoparticles have shown promising antiviral activities against different human and animal viruses, for example, both silver (AgNPs) and gold nanoparticles (AuNPs) acted as antiviral agents against human immunodeficiency virus by inhibition of the virus entry into the cells [10,11,12]. AgNPs exhibited antiviral activities against respiratory syncytial virus [13,14], hepatitis B virus [15], monkeypox virus [16], vaccinia virus [17], human parainfluenza virus [18], and herpes simplex virus [18,19]. AgNPs, silver-chitosan, iron oxide, and zinc oxide nanoparticles exhibited antiviral activity against H1N1 Influenza virus [20,21,22,23]. Copper iodide nanoparticles release reactive oxygen species (ROS), resulting in oxidation of the feline calicivirus capsid protein [24]. Copper–silver and copper–zinc nanoparticles have shown antiviral actions when tested against a bacteriophage as a model for DNA viruses [25].

The use of AgNPs in aquaculture has been reported in the last two decades [26]. Some of these investigations have focused on the use of AgNPs in water treatment, e.g., using nanoparticles in filter systems improves their efficacy [27,28]. While the rest of the publications have described the use of AgNPs in aquatic disease control, the majority of these experiments have focused on bacterial infections [29,30,31]. The main antimicrobial mechanisms of action of AgNPs include the release of Ag+ ions, ROS, adhesion to the cell membrane, interaction with microbial nucleic acids, and interference with the microbial cell signaling [32,33]. Ag+ ions could interact with thiol groups and were capable of deactivation of succinate dehydrogenase to exert their bactericidal action [34]. In spite of the several theories on AgNP’s mechanism of action in the literature, the exact molecular mechanisms of action have still not been clearly elucidated and need further investigations [34]. Up to now, very few studies have been conducted on the interaction between AgNPs and viruses in aquaculture. There is only one aquatic virus, namely the white spot syndrome virus affecting shrimp farming, against which AgNPs have been tested [35,36]. Currently, AgNPs are still not approved by the FDA as a certifiable cure. However, several products that contain AgNPs are commercially available such as Sovereign Silver and Argentyn 23, which are manufactured by Natural Immunogenics Corp. (NIC) [37].

For the present study, three different virus species isolated from fish were used. One of them is the spring viraemia of carp virus (SVCV), which has a negative-strand RNA genome belonging to the family *Rhabdoviridae* [38], and can cause significant losses, exceeding 50% mortality, particularly in yearling common carp (*Cyprinus carpio*) [39,40]. The other two chosen viruses have a dsDNA genome; however, they even belong to different taxonomic realms. The European catfish virus (ECV) is a ranavirus classified under the family *Iridoviridae* (realm *Varidnaviria*) [41], and mass mortality events are known due to this virus in populations of different catfish species (*Ameiurus melas*, *A. nebulosus*, *Silurus glanis*) [42,43,44,45,46,47,48,49]. The third virus is a herpesvirus (family *Alloherpesviridae*, realm *Duplodnaviria*) [50], namely the Ictalurid herpesvirus 2 (IcHV-2). The latter virus can also cause devastating losses in black and brown bullhead (*A. melas*, *A. nebulosus*) populations [51]. Under experimental conditions, both ECV and IcHV-2 can cause more than 90% mortality in juvenile fish stock [43,48,51].

There is no specific practical cure or effective therapy for the above-listed viral diseases. Usually, biosecurity and preventive measures are applied to fish farms in order to reduce the risk of viral infections [52]. For example, in the mid-1990s when black bullhead farming in Italy was devastated by IcHV-2, fish have been reared in farms where the water temperature could be kept below the optimal temperature for the replication of IcHV-2 (24 °C) [53]. Nevertheless, against the SVCV, several promising vaccines have been developed [54,55,56], none of which are available commercially.

To the best of our knowledge, this is the first report of the use of nanoparticles against finfish viruses.

## 2. Materials and Methods

### 2.1. Cells and Viruses

EPC (*Epithelioma Papulosum Cyprini*, ATCC CRL-2872) cells were grown in EMEM medium (Lonza, Visp, Switzerland) supplemented with 10% fetal bovine serum (FBS) (Biosera, Cholet, France), 1% HEPES buffer (1 M) (Biosera, Cholet, France), and 1% penicillin-streptomycin (Lonza, Switzerland) at 25 °C. The ECV strain (14612/2012) used in this study was isolated from brown bullhead in Hungary [49], while the IcHV-2 strain was kindly provided by Prof. Giuseppe Bovo [51], with both viruses being propagated in the EPC cell line at 25 °C, whilst the SVCV strain (ME/2020) isolated previously in our lab was propagated at 20 °C in the same cell line. The tissue culture infectious dose (TCID 50/mL) was calculated by the Reed and Muench method.

### 2.2. AgNP Synthesis

The synthesis of AgNPs was conducted by the chemical reduction method [57]. Silver nitrate (AgNO_3_) was subjected to reduction reaction with the aid of sodium citrate and sodium borohydride as reducing agents. Polyvinylpyrrolidone (PVP) was added to enhance the stability of silver nanoparticles and prevent their aggregation. The synthesized AgNPs were stored in the refrigerator at 4 ºC and covered with aluminum foil to protect from exposure to light.

### 2.3. AgNP Characterization

The synthesized AgNPs were diluted 1:10 with deionized water and imaged using transmission electron microscopy (TEM). Dynamic light scattering (DLS) was applied to measure the size distribution and zeta potential of silver nanoparticles.

### 2.4. Cytotoxicity Assay

MTT Cell Viability Assay Kit (Invitrogen, Carlsbad, CA, USA) was used to determine the cytotoxic effect of AgNPs on the EPC cells. Cells were seeded in 96-well plates (2 × 10^4^ cell/well) and incubated for 24 h at 25 °C; then, AgNPs were added in the following final concentrations: 12.5, 25, 50, and 100 ng/mL (in quadruplicates). For blanks, wells without cells were handled in the same manner as wells containing cells. Cytotoxicity was calculated as a per cent of the control.

### 2.5. In Vitro Assays for Silver Nanoparticles and Virus Interaction Studies

EPC cells were seeded in 24-well plates (2 × 10^5^ cells/well) and incubated overnight at 25 °C. Viruses (ECV and IcHV-2) at MOI of 0.01, with SVCV at MOI of 0.001, were used in the following experiments: virus pre-treatment assay, and cell pre- and post-treatment assays. AgNP was used in the following concentrations with the ECV: 12.5, 25, 50, and 100 ng/mL. Later, when the assays were carried out by the other two viruses, the 12.5 ng/mL concentration AgNP treatment was dismissed. Plates after the treatment were incubated for 48 h at 20 °C (SVCV) or at 25 °C (ECV and IcHV-2). Then, the plates were frozen at −20 °C and then proceeded with nucleic acid extraction and qPCR. All assays were performed in duplicates.

### 2.6. Virus Pre-Treatment Assay

Viruses in EMEM containing 2% FBS were incubated with different concentrations of AgNP for 1 h at 20 °C or at 25 °C depending on the virus. The virus-AgNP mixture was added to the EPC cells and incubated for 1 h; then, cells were rinsed with phosphate-buffered saline (PBS) twice, and medium with 2% FBS was added.

### 2.7. Cell Pre-Treatment Assay

EPC cells were treated with AgNP and incubated for 1 h at 20 °C or at 25 °C. The wells were then washed with PBS twice to remove free AgNPs in medium, topped up with EMEM containing 2% FBS with the virus, incubated for 1 h, and then rinsed twice with PBS. Then, medium (2% FBS) was added.

### 2.8. Cell Post-Treatment Assay

EPC cells were infected with the three viruses and incubated for 1 h at 20 °C or at 25 °C. The wells were washed with PBS twice to remove extracellular viruses, topped up with EMEM containing 2% FBS with AgNPs, and incubated for 1 h at 20 °C or at 25 °C. Then, the wells were rinsed twice and medium was added. Additionally, delayed cell post-treatment was carried out with the ECV and IcHV-2. In these assays, the AgNP treatment was applied 24 h after the virus infection.

### 2.9. Quantitative PCRs

Viral DNA and RNA were extracted by Viral Nucleic Acid Extraction Kit II (Geneaid, New Taipei City, Taiwan) according to the instructions of the manufacturer. Extracted DNA was stored at −20 °C, while the RNA was stored at −80 °C.

Quantitative real-time PCRs (qPCRs) for determining the relative amount of the viral DNA or RNA in the wells were carried out in a Bio-Rad^®^ Real-Time PCR System instrument (Bio-Rad, Hercules, CA, USA). SensiFAST^TM^ SYBR Hi-ROX Kit and SensiFAST™ SYBR^®^ Hi-ROX One-Step Kit (Bioline, London, UK) were used. The reaction mixture contained 10 µL of 2× SensiFast mix, 7.4 µL of distilled water, 0.8 µL of each primer (Table 1), and 1 µL of the target DNA in a final volume of 20 µL (in RT-PCR, 6.8 µL of water was used and it contained 0.2 µL of reverse transcriptase enzyme and 0.4 µL of RNase inhibitor). The program consisted of an initial denaturing at 95 °C for 3 min, followed by 40 cycles of 95 °C for 5 s, and 65 °C for 30 s. All qPCRs were performed in duplicates. The beta-actin gene was used as an internal standard. The results were analyzed by the Bio-Rad CFX Maestro software (Bio-Rad).

### 2.10. Statistical Analysis

Statistical analysis was performed using one-way ANOVA followed by Tukey’s post hoc test with the R Commander (version i386 4.1.1.) software. *p*-values of 0.05 or below were considered statistically significant.

## 3. Results

### 3.1. AgNP Characterisation

**Figure 1:**
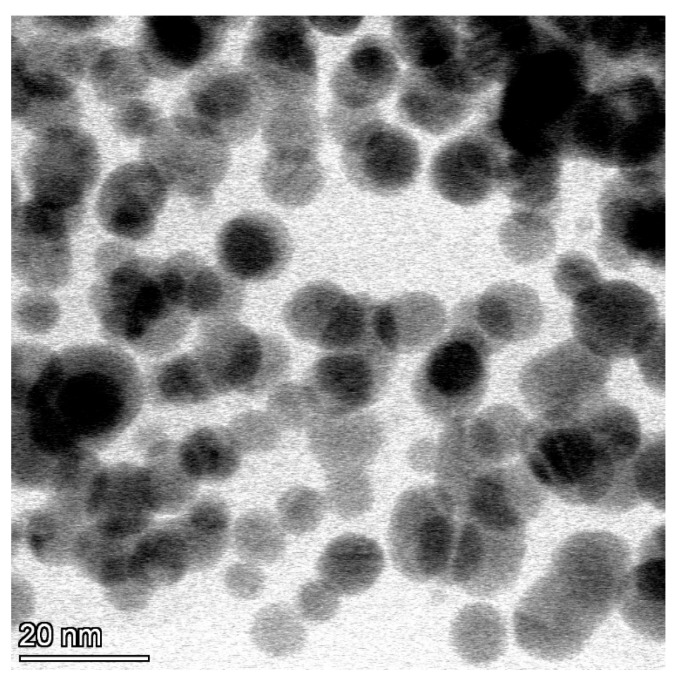
Microphotograph of silver nanoparticles with transmission electron microscopy showing the spherical shape of the particles appearing as electron-dense particles with a mean diameter of 10.2 ± 1.6 nm (scale bar = 20 nm).

TEM microphotographs of AgNPs revealed the spherical shape of the particles appearing as electron-dense particles with a mean diameter of 10.2 ± 1.6 nm (Figure 1).

**Figure 2:**
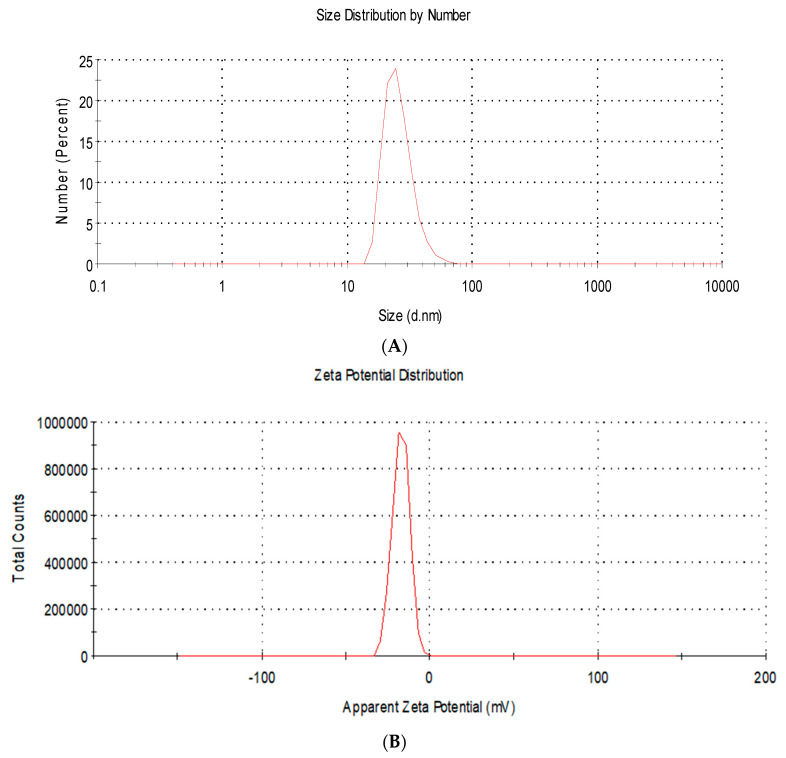
Dynamic light scattering analysis of silver nanoparticles showing (**A**) the size distribution by number with a mean diameter of 22.4 ± 5.3 nm; (**B**) the charge of AgNPs was found to be negative (−19.2 ± 2.9 mV).

Moreover, DLS analysis showed the size distribution by number with a mean diameter of 22.4 ± 5.3 nm (Figure 2A). In addition, the charge of AgNPs was found to be negative (−19.2 ± 2.9 mV) (Figure 2B).

### 3.2. Cytotoxicity Assays

The results of the MTT assay are shown in Table 2. If the difference between the control and counterpart at a certain concentration reached statistical significance, the concentration was considered to be harmful. There was no significant cytotoxic effect of AgNPs at the concentrations of 12.5 and 25 ng/mL.

### 3.3. In Vitro Assays

**Figure 3:**
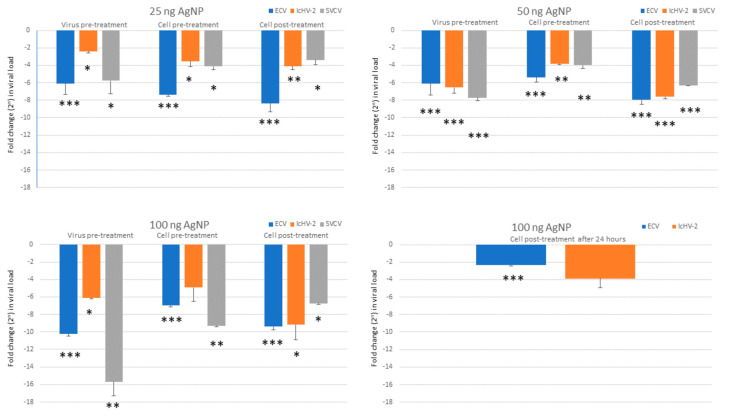
Viral load measured by qPCR, normalized against beta-actin, and calculated as ΔΔCt values. The results are represented as a fold change relative to the mean of control (virus only, non-treated cells). Values are expressed as the mean ± SEM. One-way ANOVA and Tukey’s post hoc test applied. * *p* < 0.05, ** *p* < 0.01, *** *p* < 0.001 vs. matched control.

**Figure 4:**
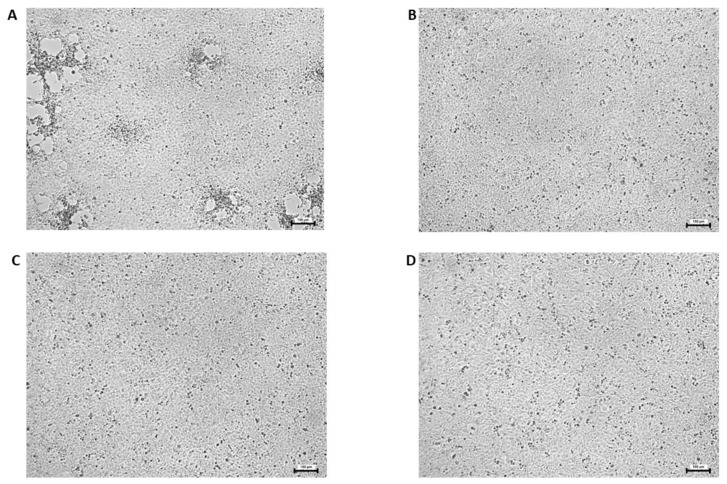
The CPE caused by the ECV 48 h after the inoculation. (**A**) Control cells (virus only), (**B**) virus pre-treatment, (**C**) cell pre-treatment, and (**D**) cell post-treatment with 25 ng/mL of AgNP. 100× magnitude.

AgNPs at 12.5 ng/mL of concentration did not reduce the viral load of ECV. Due to this result and considering that the 25 ng/mL concentration was still not cytotoxic to the cells, the 12.5 ng/mL AgNP concentration was not used with the other two viruses. The viral load of each virus examined was significantly decreased by the higher concentrations of AgNPs (25, 50, and 100 ng/mL), except one case (100 ng/mL of NP in cell pre-treatment against IcHV-2, which proved not to be significant, due to the higher SEM value). Figure 3 shows the results of the in vitro assays. A reduction in viral loads was observed in all types of treatment assays (virus pre-treatment, and cell pre- and post-treatment) (Figure 4). The 25 ng/mL concentration was not cytotoxic to the EPC cells and proved to be efficient against all three viruses, in all types of assays. At this concentration, the rate of the reduction in the viral load ranged between 70 and 330 times in the case of ECV and between 10 and 54 times in the case of SVCV. It proved to be less efficient against IcHV-2 with the 5–17-times reduction.

The cell post-treatment assays were the most effective against the ECV and SVCV; hence, with these viruses, delayed post-treatment assays (24 h) were carried out. A reduction in viral load was observed in both cases; however, the results proved to be significant only with the ECV.

## 4. Discussion

Infectious diseases caused by various pathogens such as bacteria, fungi, parasites, and viruses induce major risks to aquaculture. There are different antiparasitic and antibacterial treatments in the market for fish farms, but no direct antiviral therapy yet [1]. One of the most investigated nanoparticles in agriculture, veterinary medicine, and aquaculture is AgNPs [2]. Although the application of AgNPs in aquaculture has been reported in the past decades [26], the majority of these studies focused on the water treatment (filter system improvements) [27,28], or disease control of bacterial and protozoan infections [29,30,31]. To the best of our knowledge, this is the first report on the evaluation of the applicability of AgNPs against finfish viruses, which are known to cause devastating losses in aquaculture.

In this experiment, we studied the antiviral activity of synthesized AgNPs. To stabilize the newly formed nanoparticles, we used polyvinylpyrrolidone as a capping agent to prevent them from aggregation and agglomeration [30]. TEM photos revealed that AgNPs were electron-dense and spherically shaped particles. Their average particle size was calculated to be 10.2 ± 1.6 nm. As TEM provides information about the shape and the average size of the nanoparticles, DLS analysis is capable of showing the size distribution and particle charge. The size distribution by number showed a mean diameter of 22.4 ± 5.3 nm, which is higher than the mean calculated from TEM results due to the dispersant effect on DLS [60]. AgNPs exhibited a negative charge of −19.2 ± 2.9 mV, which reflects good stability of the synthesized particles. This negative charge is attributed to the interaction between the AgNP surface and PVP [61].

For the antiviral experiments, three very distantly related virus species were chosen intentionally. The SVCV is a –ssRNA virus causing significant economic losses in carp farming worldwide [38,39], while the other two viruses are dsDNS viruses belonging to different taxonomic realms. The IcHV-2 has been isolated from different bullhead species (Ictaluridae), while the ECV is known to cause losses in other catfish species as well (Siluridae) [42,43,44,45,46,47,48,49]. There is no effective therapy against the above-mentioned viruses. Hence, the efficacy of AgNPs was tested in vitro against these economically important fish viruses.

Firstly, the safe concentration of the AgNPs was determined in EPC cells, which proved to be 25 ng/mL. At this dose, the AgNPs decreased significantly the viral load of all three viruses in all different types of treatments (virus pre-treatment, cell pre-treatment, and cell post-treatment with the AgNPs). It proved to be the most efficient against the ECV (viral load was reduced by 70–330 times), and it was also very convincing against SVCV (10–54×). As for IcHV-2, the viral load was decreased by 5–17×, which proved to be a still significant reduction. A lower concentration of AgNPs did not prove to be efficient. Although the higher concentrations of AgNPs were harmful for the EPC cells, the in vitro experiments were also carried out in order to see whether the reductions were dose-dependent, but this was not clearly proven by the data. At a higher concentration, the AgNPs proved to be the most efficient against ECV and IcHV-2 in the cell post-treatment; hence, we tried it as a delayed post-cell-treatment experiment (AgNP treatment was applied 24 h after the virus inoculation). In these experiments, the significant reduction in the viral load of ECV is very promising. It might imply that the usage of AgNPs could be efficient therapy against ECV in vivo even after the observation of the first clinical signs of the disease.

The exact mechanism by which AgNPs execute its destroying effect on viruses is still unclear. The AgNPs might inhibit the viruses in different ways, for example, inhibition of the virus–host cell binding by preventing viral attachment or damaging the surface proteins (monkeypox virus, influenza virus, respiratory syncytial virus, herpes simplex virus, human immunodeficiency virus), inactivation of the virus prior to entry (Tacaribe virus), and interaction with the dsDNA and inhibition of viral replication (hepatitis B virus) [10,13,15,16,62,63,64,65,66]. AgNPs could bind to gp120 of HIV-1 virus, which prevents CD4-dependent virion binding and fusion [10], and they are capable of blocking the transmission of HIV-1 between infected and healthy cells [63]. In our study, we suggest that more than one mechanism occurred, since the inhibition by the AgNPs was successful in three different treatment types. The only feature common in these viruses besides that they infect finfish and cause high mortality is that the virions are enveloped. That might imply that during the virus pre-treatment, the AgNPs damage the envelope of the virions, which reduces its ability to enter into the host cells. However, in the case of the cell pre-treatment and cell post-treatment, intracellular antiviral activity must have occurred [15].

These first in vitro results against three devastating fish viruses are encouraging to continue in vivo studies and investigate the applicability and efficacy of AgNPs against viral diseases in aquaculture in the future.

## Figures and Tables

**Table 1:** Primers used in this study.

Target	Oligonucleotides	References
ECV major capsid protein gene	5′-GTT CAT GAT GCG GAT AAT GTT GT-3′ 5′-ACC TCT ACT CTT ATG CCC TCA GC-3′	[49]
SVCV glycoprotein gene	5′-TGC TGT GTT GCT TGC ACT TAT YT-3′ 5′-TCA AAC KAA RGA CCG CAT TTC G-3′	[58]
IcHV-2 DNA polymerase gene	5′-ATA CAT CGG TCT CAC TCA AGA GG-3′ 5′-TAA TGG GTA TTG GTA CAA ATC TTC ATC-3′	[59]
B-actin gene	5′-CAA CAG GGA AAA GAT GAC GCA GAT-3′ 5′-GGG AGA GCA CAG CCT GGA T-3′	this study

**Table 2:** Viability of EPC cells after one hour of exposure to different silver nanoparticle (AgNP) concentrations. * Statistically significant difference (*p* < 0.05).

Concentration of AgNP (ng/mL)	Viability ± SEM (%)
100	77.04 ± 1.70 *
50	87.21 ± 2.55 *
25	104.39 ± 2.21
12.5	99.77 ± 2.38
